# Mechanistic Insights from Human Studies in Axial Spondyloarthritis: A T Cell Story

**DOI:** 10.1007/s11926-026-01213-3

**Published:** 2026-03-05

**Authors:** Joy Um, Michael A. Paley

**Affiliations:** Department of Medicine, Washington University School of Medicine, St. Louis, MO USA

**Keywords:** Ankylosing spondylitis, Axial spondyloarthritis, HLA-B*27, T cell receptor, IL-17

## Abstract

**Purpose of review:**

Ankylosing spondylitis (AS) represents the archetype of the spondyloarthritis family defined by its strong association with HLA-B*27. While genetic susceptibility has long pointed toward adaptive immunity, the precise cellular pathways linking MHC class I alleles to axial inflammation have remained an enigma. In this article, we review the fundamental molecular and clinical evidence positioning CD8  and CD4  T cells as primary pathogenic drivers of disease in the HLA-B*27 +  patients.

**Recent findings:**

High-throughput T cell receptor (TCR) sequencing and single-cell RNA sequencing have identified “public” TRAV21/TRBV9 TCRs, that are expanded in the inflamed joints and eyes of patients. These clones initiate disease via molecular mimicry, recognizing both microbial and self-peptides presented by HLA-B*27. The clinical success of seniprutug, a monoclonal antibody selectively targeting TRBV9 +  T cells, provided the first proof-of-concept that these specific clonotypes drive clinical disease. Furthermore, high-resolution profiling has identified CD4 Th17 cells as the dominant producers of IL-17 within the synovial niche, providing a cellular basis for the efficacy of IL-17A and IL-17F blockade.

**Summary:**

AS is a disease characterized by convergent cross-reactive T cell responses. The identification of pathogenic TCR signatures and their candidate cognate antigens has moved diagnostics and management toward precision medicine.

## Introduction

Ankylosing spondylitis (AS) is a chronic inflammatory disease affecting the spinal and sacroiliac joints, resulting in chronic back pain and impaired spinal mobility. AS belongs to a group of related diseases called spondyloarthritis that share a genetic association with HLA-B*27. This disease spectrum includes reactive arthritis (ReA), psoriatic arthritis, enteropathic arthritis, and enthesitis-related juvenile idiopathic arthritis [1–10]. These conditions often feature extra-articular manifestations such as psoriasis, inflammatory bowel disease, and dactylitis. Acute anterior uveitis (AAU) is the most frequent extra-articular manifestation, especially in AS [11]. HLA-B*27 links these two conditions: one-third of AS patients develop AAU, and HLA-B*27+ patients have a fourfold higher risk of uveitis than HLA-B*27- patients [11, 12].

The genetic association between spondyloarthritis and HLA-B*27 implicated the adaptive immune system in disease susceptibility. However, defining the cellular pathophysiology has been a long-standing challenge. Recent human studies have revived the “arthritogenic peptide” model whereby HLA-B*27 presents specific peptides to pathogenic CD8 T cells to drive arthritis. In parallel, additional evidence has highlighted a Th17 CD4 T cell response as a key player in disease pathogenesis. We review the molecular and clinical evidence that positions T cells as primary drivers of HLA-B*27+  AS.

## Nomenclature in Spondyloarthritis 

Spondyloarthritis (SpA) nomenclature has evolved alongside advances in imaging. Historically, the term AS referred to chronic axial inflammation with radiographic sacroiliitis and bridging syndesmophytes. The 1984 modified New York criteria required these structural changes for diagnosis [13]. Magnetic resonance imaging (MRI) later revealed inflammatory changes that precede radiographic damage [14–16]. In response, the term axial spondyloarthritis (axSpA) was introduced in 2004 and formalized in the 2009 Assessment of SpondyloArthritis international Society (ASAS) classification criteria [14, 17, 18]. These criteria established an imaging arm, requiring sacroiliitis on radiographs or MRI, and a clinical arm, requiring HLA-B27 plus specific clinical features. Patients with radiographic sacroiliitis are classified as radiographic axSpA (r-axSpA), while others are classified as non-radiographic axSpA (nr-axSpA) via positive MRI in the imaging arm or via the clinical arm. Meta-analyses indicated that r-axSpA and nr-axSpA represent a disease spectrum with similar clinical characteristics and disease burdens [19]. Analysis of patient cohorts confirmed that r-axSpA and AS are roughly interchangeable terms, with over 90% of patients fulfilling both modified New York and ASAS criteria [20]. Consistent with the 2024 ASAS consensus statement’s recommendations [21], we will use AS and axSpA interchangeably throughout this review while adopting original source nomenclature to reflect historical context.

## Association of HLA-B*27 and AS and Potential Mechanisms

In 1973, two independent groups identified the association between AS and HLA-B*27 [22, 23]. This was one of the first associations between a common HLA allele and a complex inflammatory disease. Approximately 70–90% of patients with AS carry HLA-B*27, compared with less than 10% of the general population, suggesting antigen presentation and adaptive immunity in disease susceptibility [22, 24–28]. The strength and specificity of the association pointed toward a direct role for the HLA-B*27 molecule itself, rather than linkage disequilibrium with nearby loci, in shaping a pathogenic immune response [29]. These observations focused attention on how the structural and functional properties of HLA-B*27 might bias immune recognition in a manner that promotes chronic inflammation.

HLA-B*27 is a class I major histocompatibility complex (MHC I) molecule whose canonical function is to present peptides derived from intracellular proteins to CD8 T cells. The arthritogenic peptide hypothesis emerged in the late 1980s and early 1990s as a natural extension of advances in class I MHC biology and CD8 T cell immunology [29]. Following the demonstration that cytotoxic T lymphocyte (CTL) recognition is dependent on short peptides presented by MHC class I molecules, it became plausible that a disease-associated HLA class I allele such as HLA-B*27 could predispose to pathology by presenting a restricted set of peptides to CD8 T cells [30]. Structural studies of HLA-B*27 reinforced this idea by demonstrating that its peptide-binding groove imposes strict biochemical constraints on peptide selection with strong preference for arginine at position 2 and an extended peptide conformation [31]. Additional studies demonstrated that HLA-B*27 naturally presents defined self-derived nonameric peptides in vivo, with conserved sequence motifs consistent with structural predictions [32]. Collectively, these data distinguished HLA-B*27 from other class I alleles and suggested that HLA-B*27 presents a distinct peptide repertoire with potential to bias T cell recognition, providing a necessary foundation for models invoking autoreactive or cross-reactive CD8 T cell responses.

By the early 1990s, research distinguished the disease-associated subtypes HLA-B*27:02, B*27:04, and B*27:05 from the weakly associated or protective HLA-B*27:06 and B*27:09. These alleles differ by only a few amino acid residues within the peptide-binding groove, but with alterations sufficient to modulate peptide binding [33]. Using Epstein-Barr virus (EBV) as a model system, it was observed that disease-associated HLA-B*27 subtypes present identical immunodominant viral peptides to CD8 T cells [34]. This overlapping peptide repertoire suggested how distinct alleles could confer similar disease risk. The arthritogenic peptide hypothesis remained plausible despite minor HLA-B*27 polymorphisms through presentation of a shared set of peptides leading to T cell activation.

Two additional mechanistic models have been proposed to explain how HLA-B*27 may contribute to ankylosing spondylitis independently of classical peptide-TCR recognition. These models will be briefly summarized, as thorough reviews can be found elsewhere [35, 36]. One line of investigation arose from the observation that HLA-B*27 heavy chains can adopt noncanonical conformations at the cell surface. In the absence of β2-microglobulin, HLA-B*27 could form disulfide-bonded homodimers, dependent on the unpaired cysteine at position 67, generating a molecular species distinct from conventional HLA-B*27 heterotrimers [37]. These homodimers were detectable in vivo in patients with spondyloarthritis and were shown to engage innate immune receptors including KIR3DL1 and KIR3DL2 on subsets of T cells and NK cells [38]. Functional experiments further demonstrated that ligation of KIR3DL2 by HLA-B*27 homodimers modulates lymphocyte effector function, including inhibition of IFN-γ production, supporting a receptor-mediated pathway distinct from antigen-specific TCR engagement [39]. A related but conceptually distinct model emerged from HLA-B*27 transgenic animal studies, which showed that intrinsic misfolding of the HLA-B*27 heavy chain can induce endoplasmic reticulum stress and activation of the unfolded protein response (UPR). In HLA-B*27/human β2-microglobulin transgenic rats, macrophages accumulate misfolded heavy chains associated with BiP, leading to robust XBP1 splicing and induction of UPR-associated inflammatory programs, and these animals develop a spontaneous spondyloarthritis-like phenotype even in the absence of CD8 T cells [40, 41]. While both models provide explanations for how HLA-B*27 could promote inflammation through non-TCR-dependent mechanisms, direct evidence for sustained homodimer-driven signaling or UPR activation in human ankylosing spondylitis remains limited. Moreover, how these mechanisms would lead to tissue-specific inflammation of the sacroiliac joint or eye remains unclear.

The epistasis between ERAP1, encoding for endoplasmic reticulum aminopeptidase 1, and disease-associated HLA alleles supports the role for antigen processing and peptide presentation in AS and AAU pathogenesis. Genome-wide association studies showed that ERAP1 polymorphisms confer AS risk primarily in HLA-B*27+ individuals. However, ERAP1 epistasis also has been observed with HLA-B*40:01 in HLA-B*27- populations [42–45]. In AAU, a meta-analysis of 3,850 cases found that protective ERAP1 haplotypes reduced disease risk only in HLA-B*27+ patients (OR = 0.73, *p* = 5.2 × 10^− 10^) [46]. Together, these findings indicate that ERAP1-dependent peptide trimming may be a shared pathogenic mechanism across multiple AS-associated HLA class I alleles, reinforcing the importance of aberrant peptide presentation in disease development.

ERAP1 trims peptides to optimal length (8–10 amino acids) before their binding to MHC class I molecules. Protective variants exhibited reduced enzymatic activity in vitro [44], shifting the peptide repertoire from optimal 8-10mer to longer 10-13mer peptides with altered N-terminal residues [47, 48]. By modulating peptide length and structure, these polymorphisms may limit the presentation of autoreactive epitopes to CD8 T cells. Furthermore, ERAP1 activity was observed to affect the structural stability of HLA-B*27 molecule. AS patients with protective variants showed reduced surface expression of HLA-B*27 free heavy chains (FHC) which resulted in lower Th17 expansion and IL-17 A secretion [49]. In HLA-B*27 transgenic rats, ERAP1 knockout reduced HLA-B*27 misfolding and suppressed the unfolded protein response, mitigating *Il-23a* upregulation and decreasing arthritis prevalence [50]. These findings illustrate the complex influence of HLA-B*27 on disease biology, highlighting a dual role in structural stability and antigen presentation that converges on the activation of T cells.

## Diversity of TCR and VDJ Recombination

To understand the recent advances in T cell biology in AS that have established T cells as primary drivers of disease, we will first review how T cells recognize peptide-MHC and how T cell receptor (TCR) diversity is generated. During thymic development, each T cell acquires a unique TCR, with approximately 95% of circulating T cells expressing αβ TCRs [51]. The TCR α chain is generated through recombination of variable (V) and joining (J) gene segments, whereas the β chain incorporates diversity (D) segments in addition to V and J. Somatic recombination of these gene segments, together with imprecise junctional processing, generates a diverse TCR repertoire that enables recognition of a vast array of antigens while maintaining self-tolerance. Theoretical estimates suggest that more than 10^15^ distinct αβ TCRs may be generated through the V(D)J recombination in humans, underscoring the importance of TCR diversity in adaptive immunity [52]. Disruption of this process leads to profound immunodeficiency states, such as severe combined immunodeficiency, highlighting its biological necessity to form proper defense against pathogens.

While the theoretical diversity of the αβ TCR repertoire is vast, the actual functional repertoire in an individual is significantly smaller, estimated at approximately 10^7^ to 10^8^ unique clonotypes at any given time [53]. This numerical constraint suggests that the immune system cannot possibly maintain a unique TCR for every potential pathogen. Therefore, the system relies on TCR cross-reactivity, where a single receptor can recognize multiple structurally related peptides.

Antigen recognition by the TCR is mediated through six complementarity-determining regions (CDRs), with each α and β chain contributing CDR1, CDR2, and CDR3 loops. CDR1 and CDR2 are germline-encoded by the V gene segment and typically interact with conserved regions of the MHC molecule, whereas the CDR3 sequence is generated by V(D)J recombination and usually makes dominant contacts with the bound peptide. As a result, CDR3 amino acid sequences function as molecular “barcodes” that reflect antigen-driven selection within the T cell repertoire.

This structural and genetic organization provides the basis for modern analyses of T cell involvement in human disease. Sequencing of TCR CDR3 regions has become a powerful tool for interrogating antigen-selected T cell populations across autoimmunity, cancer, and infection, allowing direct inference of clonal expansion, repertoire bias, and shared antigen specificity. In the context of AS, these principles form the conceptual foundation for interpreting how HLA-B*27-restricted peptide presentation shapes pathogenic T cell responses, which will be examined in detail in subsequent sections.

## Role of CD8 T Cells in AS

### Public T Cell Clones First Discovered in Reactive Arthritis

The first discovery of public and clonally expanded T cells came from another member of the spondyloarthritis family, reactive arthritis (ReA). TCRβ analysis of cultured T cell clones tested the arthritogenic peptide hypothesis by asking whether there was an oligoclonal expansion in the inflamed joints of ReA (*n* = 4) and AS (*n* = 3) patients to suggest an in situ antigen-driven immune response [54]. Spectratyping revealed oligoclonal expansion in synovial fluid not seen in peripheral blood, defined by restricted BV-BJ usage and discrete CDR3 lengths, consistent with local antigen-driven selection of these T cells rather than systemic activation.

Sequencing of dominant synovial expansions revealed presence of the same CDR3β amino acid sequence (SVGLYSTDTQ) across two distinct BV families (BV1 and BV23), along with other closely related variants differing by only one or two amino acid residues (Table 1). The similar CDR3β sequences with different V genes argued for strong selection at the level of peptide recognition. Additional shared motifs (SVGLFSTDTQ and SVGDYSTDTQ) were also present across BV1 and BV23 expansions, consistent with the concept of convergent solutions to a common antigenic constraint.

Furthermore, identical CDR3β amino acid sequences were observed across two different patients with ReA triggered by distinct organisms (*Yersinia enterocolitica* in one patient, unknown organism in the other patient). Nucleotide-level analysis showed different nucleotide sequences encoding the same amino acid sequences, providing direct evidence of convergent recombination towards a shared specificity. One of these motifs had previously been reported in a *Salmonella*-triggered ReA patient, suggesting that various inflammatory triggers elicited a convergent T cell response to a shared antigen [60]. Importantly, these expanded clones were found within the CD8+ CD45RO+ memory compartment, suggesting recognition of peptides presented by MHC Class I proteins, such as HLA-B*27.

The conserved CDR3β motifs represented a disease-specific adaptive immune response, rather than inflammation-associated repertoire skewing [55]. Subsequent comparison of BV1-BJ2S3 compartment across cohorts comprised of patients with acute and chronic ReA, AS, and rheumatoid arthritis revealed dominant BV1-BJ2S3 expansions with conserved amino acid sequences only in HLA-B*27+ ReA synovial fluid. These motifs were absent in AS, rheumatoid arthritis, and healthy controls. A systematic database search revealed that a large fraction of published HLA-B*27-associated TCRβ sequences clustered around a single canonical motif: BV1/23-CASSVG(V/I/L)(Y/F)STDTQYF-BJ2S3) (Table 1). Identical or near-identical sequences appeared across different ReA patients, laboratories, and triggering pathogens. Even with the technical limitations of early Sanger-based TCR sequencing, observation of (i) identical CDR3β sequences arising from different BV genes within a patient, (ii) the presence of public CDR3β sequences shared across multiple patients, and (iii) disease-specific enrichment of a conserved CDR3β motif provided molecular evidence that HLA-B*27-associated arthritis involves antigen-driven, convergent CD8 T cell responses. At the time, these findings in ReA did not generalize to AS, likely due to the limited depth of early repertoire sampling.

### High-throughput TCR Sequencing in AS

High-throughput TCRβ sequencing allowed a re-evaluation of convergent CD8 T cell responses in spondyloarthritis. This technology overcame the depth and sampling limitations of spectratyping and Sanger-based methods. One study profiled peripheral blood TCRβ repertories in 191 HLA-B*27+ AS patients, 43 HLA-B*27- AS patients, and 227 controls [56]. Analysis of 77 million TCRβ clonotypes identified a set of CDR3β amino acid motifs enriched in HLA-B*27+ AS. Two motifs remained enriched when comparing HLA-B*27+ AS patients to HLA-B*27+ healthy controls, indicating disease specificity independent of HLA allele.

The strongest association matched the established ReA CDR3β motif CASSVG(V/I/L)(Y/F)STDTQYF, flanked by TRBV9 and TRBJ2.3 (Table 1). This shared motif provided the first molecular bridge between infection-triggered ReA and AS, showing that shared CD8 T cell features persist across HLA-B*27 +  spondyloarthritis spectrum. Most motif-bearing clonotypes localized to the CD8 compartment, consistent with an HLA-B*27-restricted mechanism and class I-restricted antigen presentation.

Later analyses of paired peripheral blood and synovial fluid provided spatial context for these motifs. CD8 TCRβ clonotypes containing the conserved STDTQYF core were enriched in AS joint fluid relative to blood and were absent from healthy controls [61]. Another study corroborated this interpretation by detecting identical public CD8 TCRβ clonotypes in inflamed synovium, including the canonical motif CASSVGLFSTDTQYF associated with TRBV9 [62] (Table 1). Together, these studies connected the shared CDR3β sequences from the peripheral blood to the inflamed joints, unifying ReA and AS through convergent CD8 T cell receptor usage. However, because these analyses relied on β chain sequencing only, the identity of the paired α chain and the cognate peptides presented by HLA-B*27 remained unresolved.

### Discovery of Candidate Cognate Antigens in AS Through Paired TCRαβ Sequencing

The development of paired single-cell RNA and TCR sequencing resolved the antigen specificity of CD8 T cells in HLA-B*27-related disease. Analysis of blood, synovial fluid, and ocular fluid in separate AS and AAU cohorts showed that the public TRBV9-Y/FSTDTQ-TRBJ2.3 β-chain paired with a single α-chain variable region, TRAV21 [57, 58] (Table 1). This αβ pairing persisted across tissues, patient cohorts, and continents. Moreover, these T cell clones exhibited 10- to 100-fold enrichment in inflamed joints and eyes compared to peripheral blood, suggesting antigen-specific recruitment to sites of inflammation. The presence of this identical TCR signature in the eye of an AAU patient without clinical AS indicated a shared pathogenic mechanism between AS and AAU.

To identify cognate antigens, AS- and AAU-derived TCRs served as molecular probes to screen HLA-B*27:05 yeast display peptide libraries. The TRAV21/TRBV9 receptors recognized both microbial peptides and self-peptides derived from proteins including GPER1, PRPF3, and RNASEH2B [58]. Among the autoantigens, GPER1 was a primary candidate due to its broad activation of AS- and AAU-derived TCRs and its expression in the cartilage, bone, and eye—tissues targeted by AS and AAU [63–66]. Among the microbial antigens, YeiH activated multiple AS- and AAU-derived TCRs. YeiH peptide is a conserved inner-membrane protein present across Gram-negative bacteria, including *Salmonella*,* Shigella*,* Campylobacter*,* Yersinia enterocolitica*,* Escherichia coli*, and *Klebsiella* [67]. *Salmonella*, *Shigella*,* Campylobacter*,* and Yersinia* are well-established triggers for HLA-B27-associated ReA [68], while *Escherichia coli* and *Klebsiella* also may act as enteric triggers [69, 70].

**Fig. 1:**
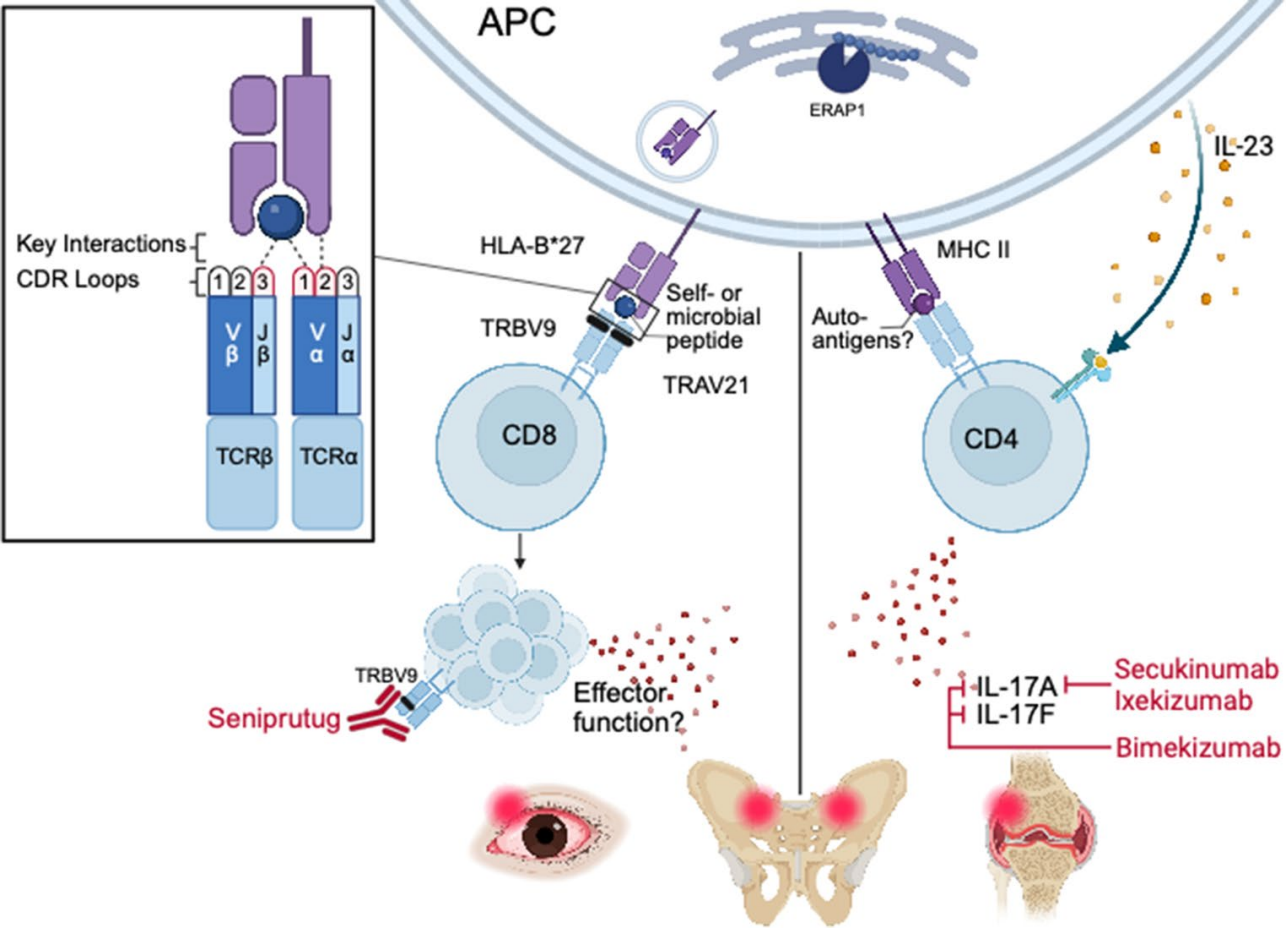
Mechanisms of T cell-driven inflammation in HLA-B*27-associated disease. Peptides derived from microbes (e.g., YeiH) or self-proteins (e.g., GPER1, PRPF3, RNASEH2B) undergo processing by ERAP1 in antigen presenting cells (APC) for presentation by HLA-B*27 to CD8 T cells. Pathogenic CD8 T cells use TRAV21/TRBV9 receptors to recognize shared peptide-MHC motifs. This recognition leads to clonal expansion and tissue-localized inflammation in the eyes (AAU) and joints (AS/axSpA). Structurally, CDR3β loop contacts peptide positions P5-P8, while CDR1α engages positions P4-P5. The CDR2α loop facilitates MHC-specificity through contacts with HLA-B*27

Structural analyses demonstrated a conserved mode of engagement that allows TCR cross-reactivity between these microbial and self-peptides despite varied primary amino acid sequences. Crystal structures of TRAV21/TRBV9 TCRs bound to peptide-HLA-B*27:05 complexes showed a uniform docking orientation and footprint regardless of peptide identity. A conserved bulky residue within the CDR3β loop (Tyr98 or Phe98) inserted into a defined pocket on the peptide-MHC surface to contact peptide positions 5 through 8. Germline-encoded residues within CDR1α engaged positions 4 and 5, while CDR2α mediated MHC-specificity through dense contacts with HLA-B*27 (Fig. 1). Thus, TRAV21 encodes for both peptide and HLA-B*27 recognition, providing a mechanism for universal usage of this V gene segment in AS- and AAU-associated TCRs.

These data also suggested a rationale for HLA-B*27 subtype-specific disease risk: the self-peptide GPER1 elicited robust T cell activation when presented by disease-associated HLA-B*27:05 allele, but not by the protective HLA-B*27:09 allele. The D116H substitution in HLA-B*27:09 introduces steric constraints that impair presentation of pathogenic peptides with certain residues (such as arginine at P9). In contrast, both HLA-B*27:05 and HLA-B*27:09 could present the bacterial peptide YeiH and activate AS- and AAU-associated TCRs, suggesting that presentation of autoantigens, rather than microbial antigens, drives the development of spondyloarthritis. Defining the structural basis of molecular mimicry enabled the selective therapeutic targeting of the cross-reactive T cell clones.

### From Disease-Associated TCR To Pathogenic TCR

Targeted depletion of TRBV9+ CD8 T cells provided evidence for the pathogenicity of these clonotypes in HLA-B*27-related disease. An HLA-B*27+ patient with severe refractory AS received the monoclonal antibody seniprutug (BCD-180) selectively targeting the TRBV9 segment of the TCRβ chain [71]. This intervention resulted in the rapid depletion of TRBV9+ CD8 T cells from peripheral blood while maintaining the remainder of the T cell repertoire. Improvement in disease activity allowed patient to stop systemic immunosuppression in the form of a TNF inhibitor. These results suggest that TRBV9+ clonotypes act as active drivers of disease rather than passive bystanders.

A phase 2 clinical trial involving 260 HLA-B*27+ patients with active radiographic axial spondyloarthritis further evaluated the efficacy of seniprutug [72]. TRBV9 depletion resulted in higher Assessment in Spondyloarthritis International Society 40% response (ASAS40) rates compared with placebo at 24 weeks. Improvement also occurred in disease activity scores such as Axial Spondyloarthritis Disease Activity Score (ASDAS-CRP), Bath Ankylosing Spondylitis Disease Activity Index (BASDAI), Bath Ankylosing Spondylitis Functional Index (BASFI), and MRI inflammation scores. Safety and immunogenicity profiles did not differ from the placebo group. Pharmacodynamic analysis confirmed sustained depletion of TRBV9+ T cells, linking target engagement with clinical efficacy. These data identified TRBV9+ T cells as a potential therapeutic target in HLA-B*27-associated spondyloarthritis.

Despite the evidence of therapeutic promise of TRBV9-depleting therapy, questions remain regarding the generalizability of such targeted treatment strategy across all HLA-B*27-associated diseases. In AAU patients, pathogenic CD8 T cells used alternative TRBVs to TRBV9, such as TRBV5-5, which is consistent with the multiple TRBVs identified in the earliest studies of ReA [54, 59] (Table 1). Re-analysis of four AAU patients suggested that TRBV9-directed therapy would deplete the pathogenic repertoire in only one individual [59]. The remaining three patients possessed antigen-reactive clonotypes that used alternative TRBV genes, which could undergo compensatory clonal expansion following TRBV9 depletion. While TRBV9-targeting served as a proof-of-concept for precision immunotherapy, targeting the TRAV21+ population may offer a more comprehensive therapeutic approach. However, such strategies would require evaluation of safety and off-target effects.

## Role of CD4 T Cells in AS

While the association with HLA-B*27 traditionally prioritized a CD8 T cell model, the disease-specific functions of pathogenic CD8 T cells remain unknown. Genome-wide association studies (GWAS) have implicated CD4 T cells as another driver of disease. Genetic analyses identified single nucleotide polymorphisms (SNPs) in the IL-23 receptor (*IL23R*) and the p40 subunit of IL-23 (*IL12B*), establishing the IL-23/IL-17 signaling pathway in AS [42, 44]. These findings suggested that Th17 cell development and cytokine production drive chronic inflammation in parallel with MHC Class I-restricted antigen presentation.

Clinical trials with IL-17 blockade in axSpA demonstrated the pathogenicity of this CD4 T cell-driven axis. Secukinumab, a monoclonal antibody targeting IL-17A, improved ASAS20 and ASAS40 response rates in several randomized trials [73]. Ixekizumab, another IL-17A inhibitor, showed similar efficacy in both biologic-naïve and TNF-inhibitor-experienced cohorts [74]. Dual inhibition of IL-17A and IL-17F by bimekizumab produced clinical responses and objective reductions in MRI-detected inflammation. These outcomes identify Type 17 cytokines as key effectors [75].

Mechanistic studies have pointed to CD4 T cells as a major source of pathogenic IL-17. These cells are enriched in the inflamed synovium compared to peripheral blood of spondyloarthritis patients and serve as the dominant cellular source of IL-17, suggesting local expansion or recruitment [76, 77]. Of note, these IL-17+ CD4+ T cells frequently express Killer Cell Immunoglobulin-like Receptors (KIR), particularly KIR3DL2, which may link their activation back to the HLA-B*27 homodimer model [77]. The engagement of KIR3DL2 by B27 homodimers could potentially promote Th17 survival and effector function, providing a mechanistic bridge where genetic risk factors and cytokine-driven inflammation converge on the CD4 T cells.

Additional reports suggest that the source of IL-17 may extend into the CD8 compartment. Recent high-resolution single-cell studies in spondyloarthritis have identified specific CD8 clusters with phenotypic and transcriptomic profiles that overlap with Th17 programs [78]. How this “Tc17” phenotype, previously described in conditions like multiple sclerosis [79, 80], connects to the antigen-specificity of the cross-reactive CD8 T cells remains to be defined.

## Future Directions and Remaining Questions

Characterizing the developmental trajectory and effector programs of the pathogenic T cell clones will clarify how these populations sustain chronic inflammation. Future therapeutic strategies may need to move beyond single TCR segment depletion toward alternative targets associated with pathogenic T cells, such as the use of PD-1 agonists to re-establish exhaustion in these activated effector populations. The generalizability of T-cell-directed therapies across diverse HLA-B*27 suballeles, such as B*27:04, also remains an open question.

TCR sequencing is transitioning from a discovery tool to a clinical diagnostic. High-throughput sequencing of the T cell repertoire can predict disease states, offering a molecular window into subclinical progression and therapeutic response [81]. These advancements suggest that immune repertoire monitoring will be central to precision medicine in HLA-B*27-associated diseases and beyond (Fig. 1 and Table 1).

**Table 1:** Summary of pathogenic CDR3β motifs in HLA-B*27-associated diseases

Study reference	Disease context	Key CDR3β motif/sequence	Vβ/Jβ gene usage	Significance of finding
Dulphy et al. (1999) [54]	ReA (synovial fluid)	CASS(V/P)G(L/D)(Y/F)STDTQ	TRBV1/TRBJ2.3 TRBV23/TRBJ2.3	First evidence of oligoclonal T cell expansion and presence of public clones in ReA synovial fluid
May et al. (2002) [55]	ReA vs. AS vs. rheumatoid arthritis (synovial fluid)	CASS(V/P)G(V/I/L/D)(Y/F)STDTQ	TRBV1/TRBJ2.3 TRBV23/TRBJ2.3	Established motifs as ReA-specific signature
Faham et al. (2017) [56]	AS (peripheral blood)	CASSVG(V/I/L)(Y/F)STDTQ	TRBV9/TRBJ2.3	ReA-associated motif significantly enriched in blood of AS patients
Komech et al. (2018) [57]	AS (synovial fluid)	CASS(V/L)G(V/L)(Y/F)STDTQ	TRBV9/TRBJ2.3	Disease-associated motif enriched in joint fluid over blood in AS
Yang et al. (2022) [58]	axSpA, AAU (synovial fluid, ocular fluid, peripheral blood)	CASS(V/L)G(L/T)(Y/F)STDTQ	TRBV9/TRBJ2.3	Disease-associated motif enriched in inflamed eye and joint over blood in AAU and AS, respectively. Resolved paired TRAV21 usage.
Paley et al. (2024) [59]	AAU (ocular fluid)	CASS(V/L/F/T/S/P)(G/A)(L/T)(Y/F)STDTQ	TRBV9 TRBV5-5 TRBV5-4 TRBV7-3	TRBV9 dispensable for antigen recognition

## Conclusions

Five decades of investigation into the immunopathogenesis of AS have transitioned the field from the initial observation of HLA-B*27 association to a mechanistic understanding of T cell-driven pathology. Recent data supported a shift in therapeutic strategies from broad cytokine blockade toward precision T cell depletion. The convergence of high-throughput repertoire sequencing, paired TCRαβ reconstruction, and the clinical success of cytokine-targeted therapies have positioned both CD8 and CD4 T cells as primary drivers of axial inflammation. As our understanding of these antigen-defined populations deepens, the focus of AS/axSpA management allows for molecularly targeted immune reprogramming to replace systemic immunosuppression.

## Key References

- Wellcome Trust Case Control C, Australo-Anglo-American Spondylitis C. Association scan of 14,500 nonsynonymous SNPs in four diseases identifies autoimmunity variants. Nat Genet. 2007;39 [11]:1329-37.
  - ○ Identified the non-MHC susceptibility loci ERAP1 and IL23R in AS. These findings establish the genetic basis for the IL-23/IL-17 axis and its interaction with HLA-B*27.
- Gelfman S, Moscati A, Huergo SM, Wang R, Rajagopal V, Parikshak N, et al. A large meta-analysis identifies genes associated with anterior uveitis. Nat Commun. 2023;14 [1]:7300.
  - ○ Confirmed that ERAP1 variants modify AAU risk only in HLA-B*27+ patients. This data support a model where shared peptide processing drives both axial and ocular inflammation.
- Dulphy N, Peyrat M-A, Tieng V, Douay C, Rabian C, Tamouza R, et al. Common Intra-Articular T Cell Expansions in Patients with Reactive Arthritis: Identical β-Chain Junctional Sequences and Cytotoxicity Toward HLA-B27. J Immunol. 1999;162:3830-9.
  - ○ Provided one of the first evidence of conserved, “public” TCRβ amino acid sequences expanded in the synovial fluid of reactive arthritis patients. Established that spondyloarthritis involves a convergent, antigen-driven T cell response.
- Faham M, Carlton V, Moorhead M, Zheng J, Klinger M, Pepin F, et al. Discovery of T Cell Receptor beta Motifs Specific to HLA-B27-Positive Ankylosing Spondylitis by Deep Repertoire Sequence Analysis. Arthritis Rheumatol. 2017;69 [4]:774 − 84.
  - ○ Identified TCRβ motifs enriched in HLA-B*27+ AS patients via high-throughput sequencing. Demonstrated that T cell motifs found in reactive arthritis also occur in the broader AS population.
- Zheng M, Zhang X, Zhou Y, Tang J, Han Q, Zhang Y, et al. TCR repertoire and CDR3 motif analyses depict the role of αβ T cells in ankylosing spondylitis. EBioMedicine. 2019;47:414 − 26.
  - ○ Demonstrated that specific αβ T cell motifs are expanded within the synovial fluid, providing evidence that these T cells participate in local tissue inflammation.
- Yang X, Garner LI, Zvyagin IV, Paley MA, Komech EA, Jude KM, et al. Autoimmunity-associated T cell receptors recognize HLA-B*27-bound peptides. Nature. 2022;612:771-9.
  - ○ Identified TRAV21/TRAV9 receptor as the “public” TCR signature shared across patients with AS and AAU. Identified both microbial (YeiH) and self-peptides (GPER1) as cognate antigens, providing a structural basis for molecular mimicry in spondyloarthritis.
- Britanova OV, Lupyr KR, Staroverov DB, Shagina IA, Aleksandrov AA, Ustyugov YY, et al. Targeted depletion of TRBV9(+) T cells as immunotherapy in a patient with ankylosing spondylitis. Nat Med. 2023;29 [11]:2731-6.
  - ○ Suggested TRBV9 + T cells as active drivers of disease by demonstrating that selective depletion of these cells using a monoclonal antibody led to clinical remission in a patient with severe AS.
- Liu F, Shi H, Turner JD, Anscombe R, Li J, Sekine T, et al. CD4 + tissue-resident memory Th17 cells are a major source of IL-17 A in Spondyloarthritis synovial tissue. Ann Rheum Dis. 2025;84 [7]:1151-63.
  - ○ Identified tissue-resident memory Th17 cells as primary source of synovial IL-17 A, defining cellular basis for the efficacy of IL-17 inhibitors in spondyloarthritis.

## Data Availability

No datasets were generated or analysed during the current study.
